# Effect of Thyroid Hormone Therapy on Fatigability in Older Adults With Subclinical Hypothyroidism: A Nested Study Within a Randomized Placebo-Controlled Trial

**DOI:** 10.1093/gerona/glaa123

**Published:** 2020-06-06

**Authors:** Mirah J Stuber, Elisavet Moutzouri, Martin Feller, Cinzia Del Giovane, Douglas C Bauer, Manuel R Blum, Tinh-Hai Collet, Jacobijn Gussekloo, Simon P Mooijaart, Vera J C McCarthy, Drahomir Aujesky, Rudi Westendorp, David J Stott, Nancy W Glynn, Patricia M Kearney, Nicolas Rodondi

**Affiliations:** 1 Institute of Primary Health Care (BIHAM), University of Bern, Switzerland; 2 Department of General Internal Medicine, Inselspital, Bern University Hospital, Switzerland; 3 Departments of Medicine and Epidemiology and Biostatistics, University of California, San Francisco; 4 Department of Health Research and Policy, Division of Epidemiology, Stanford University School of Medicine, California; 5 Service of Endocrinology, Diabetes, Nutrition and Therapeutic Education, Geneva University Hospitals, Switzerland; 6 Department of Internal Medicine, Section Gerontology and Geriatrics, Leiden University Center, the Netherlands; 7 Department of Public Health and Primary Care, Leiden University Center, the Netherlands; 8 School of Nursing and Midwifery, University College Cork, Ireland; 9 Department of Public Health and Center for Healthy Aging, University of Copenhagen, Denmark; 10 Institute of Cardiovascular Medicine, University of Glasgow, Scotland; 11 Department of Epidemiology, Center for Aging and Population Health, Graduate School of Public Health, University of Pittsburgh, Pennsylvania; 12 School of Public Health, University College Cork, Ireland

**Keywords:** Levothyroxine, Fatigue, Thyroid disease

## Abstract

**Background:**

Fatigue often triggers screening for and treatment of subclinical hypothyroidism. However, data on the impact of levothyroxine on fatigue is limited and previous studies might not have captured all aspects of fatigue.

**Method:**

This study is nested within the randomized, placebo-controlled, multicenter TRUST trial, including community-dwelling participants aged ≥65 and older, with persistent subclinical hypothyroidism (TSH 4.60–19.99 mIU/L, normal free thyroxine levels) from Switzerland and Ireland. Interventions consisted of daily levothyroxine starting with 50 μg (25 μg if weight <50 kg or known coronary heart diseases) together with dose adjustments to achieve a normal TSH and mock titration in the placebo group. Main outcome was the change in physical and mental fatigability using the Pittsburgh Fatigability Scale over 1 year, assessed through multivariable linear regression with adjustment for country, sex, and levothyroxine starting dose.

**Results:**

Among 230 participants, the mean ± standard deviation (*SD*) TSH was 6.2 ± 1.9 mIU/L at baseline and decreased to 3.1 ± 1.3 with LT4 (*n* = 119) versus 5.3 ± 2.3 with placebo (*n* = 111, *p* < .001) after 1 year. After adjustment we found no between-group difference at 1 year on perceived physical (0.2; 95% CI −1.8 to 2.1; *p* = .88), or mental fatigability (−1.0; 95% CI −2.8 to 0.8; *p* = .26). In participants with higher fatigability at baseline (≥15 points for the physical score [*n* = 88] or ≥13 points for the mental score [*n* = 41]), the adjusted between-group differences at 1 year were 0.4 (95% CI −3.6 to 2.8, *p* = .79) and −2.2 (95% CI −8.8 to 4.5, *p* = .51).

**Conclusions:**

Levothyroxine in older adults with mild subclinical hypothyroidism provides no change in physical or mental fatigability.

Subclinical hypothyroidism (SHypo), defined as the presence of an elevated serum thyrotropin (TSH) level combined with free thyroxine (fT4) level in the normal range (1), affects between 8% and 18% of adults aged 65 years or older, with a higher prevalence among women (2). SHypo is either asymptomatic or can have symptoms similar to those observed in overt hypothyroidism. Among these, global fatigue (or tiredness, which will be used as synonym in the following) is one of the most common causes for thyroid hormone testing in general practice and often results in therapy (3–5). Nevertheless, evidence from randomized controlled trial investigating the effect of levothyroxine (LT4) replacement on fatigue in SHypo is very limited, with conflicting previous data (6–8). The TRUST trial (“Thyroid Hormone Therapy for Older Adults with Subclinical Hypothyroidism”) is the only trial providing quantitative data for tiredness as outcome and is the largest, randomized, multicenter trial with the power to detect clinically meaningful benefits from LT4 replacement in older adults with SHypo (6). Its findings indicated no effect of LT4 replacement on global fatigue, as assessed by the Thyroid Related Quality-of-Life Patient-Reported Outcome measure (ThyPRO) (6,9). However, assessing global fatigue without taking activity into account might result in misleading conclusions since older adults, in particular, tend to adjust their activity levels in such a way that perceived fatigue remains tolerable (10). Assessing fatigability, a concept which anchors tiredness to a set of activities of defined intensity and duration, has proven to be a more sensitive measure for detecting fatigue rather than using global fatigue scores (10). In view of the remaining controversy on the benefit of LT4 replacement in SHypo regarding symptoms (11,12), this pre-registered study nested within the TRUST trial aimed at extending the TRUST trial findings with this novel concept of fatigability using the Pittsburgh Fatigability Scale (PFS) (10,13).

## Method

This study is registered on ClinicalTrials.gov (number NCT02500342) as a nested study from the TRUST trial performed in Scotland, Ireland, the Netherlands, and Switzerland (6). This nested study was designed after the recruitment of the main trial started since the scale used for outcome measure was published afterward (January 2015) (13).

This nested study included the TRUST participants included at the two Swiss study centers (Inselspital, Bern University Hospital, and Centre Hospitalier Universitaire Vaudois, Lausanne University Hospital) and the Irish center (University College Cork, National University of Ireland, Ireland) (14). The trial was approved by the relevant ethics committees, and participants provided written informed consent.

### The TRUST Trial

As previously published (14), the TRUST trial was a multicenter randomized, placebo-controlled parallel-group trial that included community-dwelling adults aged ≥ 65 years with untreated SHypo. In brief, participants were assigned to treatment or placebo group through permuted block randomization in a 1:1 ratio with stratification for sex, country, and starting dose. SHypo was defined as the presence of persistently elevated TSH levels (4.6–19.9 mIU/L) with fT4 within the assay reference range. TSH levels counted as persistently elevated if increased at a minimum of two occasions at least 3 months apart, over a maximum period of 3 years. The intervention consisted of daily LT4 doses, starting with 50 μg (or 25 μg in participants with bodyweight < 50 kg or with known coronary heart diseases, that is, symptoms of angina pectoris, or previous myocardial infarction), followed by dose adjustments to achieve a TSH level within the reference range (6). Blinding of participants was ensured by matching LT4 and placebo tablets as well as mock titrations in the placebo group, and blinding of clinicians and study centers through remote laboratory analysis of blood samples for TSH and dose titration as per computer algorithm.

The study was funded by the European Union FP7 and by the Swiss National Science Foundation (SNSF) for this nested study. LT4 as well as matching placebo were supplied free of charge by Merck KGaA (Darmstadt, Germany). The funders, sponsors (NHS Greater Glasgow and Clyde Health Board and University of Glasgow, United Kingdom; University College Cork, Ireland; Leiden University Medical Center, the Netherlands; and University of Bern and Bern University Hospital, Switzerland) and Merck had no influence on the main and nested studies’ designs, analyses or reporting.

The primary outcomes of this study were defined prior to data collection as the mean follow-up scores for mental and physical fatigability, measured by the PFS (13), after 1 year of follow-up with adjustment for baseline scores. Exploratory secondary outcome measures (that were not pre-specified) were the number of participants reporting higher physical (≥15 points) or mental fatigability (≥13 points) after 1 year of treatment with adjustment for baseline numbers, based on previously established cut points (15).

### Pittsburgh Fatigability Scale

The PFS is a reliable, sensitive scale, and the first-validated self-report tool to measure perceived fatigability in older adults (13). It is a 10-item questionnaire asking the participants to estimate the physical and mental fatigability they expect or image they would have after performance of different activities of specific intensity and duration. The two independent subscores (physical and mental) range from 0 to 50, with higher scores indicating greater fatigability. The cut points for higher fatigability are set at ≥15 points for the physical PFS subscore and at ≥13 points for the mental PFS subscore (15,16). The minimal clinically important difference is estimated at 2 to 3 points (13,17). The PFS has been translated into German and French, including two forward translations, synthesis of translations, and two back-translations.

### Statistical Analysis

We performed the main analysis in a modified intention-to-treat population, which included all participants with outcome of interest, and not more than three missing answers in the PFS. For up to three missing items, we imputed data using an imputation method published by the scale’s developers (18). Imputed data were based on the mean value of an individual’s complete responses with adjustments for varying intensity levels of the different activities, sex differences as well as differences in reported fatigability levels between participants who have versus those who have not done each specific activity (18). Multivariable linear regression was conducted for the physical and mental PFS scores at 1 year with adjustment for the stratification variables used for the randomization (country, sex, starting dose of LT4), as well as for corresponding PFS baseline values. Goodness of model fit was assessed through distribution plots of residuals and scatter plots of standardized residuals against predicted values.

The between-group difference in number of participants with higher fatigability at follow-up was assessed using multivariable logistic regression adjusting for the same variables as described above.

We conducted several sensitivity analyses: (a) in a population limited to participants with complete outcome data (participants who have answered to all the questions from baseline and follow-up questionnaire), (b) using the inverse probability weighting method in order to adjust for a possible attrition bias due to loss to follow-up (12 months missing primary outcomes) (19); the covariates included in the logistic regression model to estimate inverse probability weightings were chosen based on clinical judgment: age, body-mass index, country, sex and the number of comorbidities, (c) in participants who, after 1 year, adhered to the trial regimen in accordance with the protocol (per-protocol population), (d) only in participants who reported higher fatigability at baseline, (e) two exploratory sensitivity analyses, one excluding participants with diabetes and one adjusting for the presence of diabetes, to adjust for the baseline imbalance of diabetes prevalence between the groups, (f) an analysis of square-root transformed data to account for skewness in baseline scores, (g) in participants with TSH levels in the upper quartile.

Power calculation (Analysis of covariance [ANCOVA] method) for 110 participants per allocation group assuming a standard deviation (*SD*) of 9 in baseline fatigability and a baseline to follow-up correlation of 0.7 resulted in a 93.3% power to detect a difference of three points (minimal clinically important difference) in the mean follow-up fatigability scores at a two-sided alpha level of 0.05 (20).

## Results

### Trial Population

**Figure 1.**
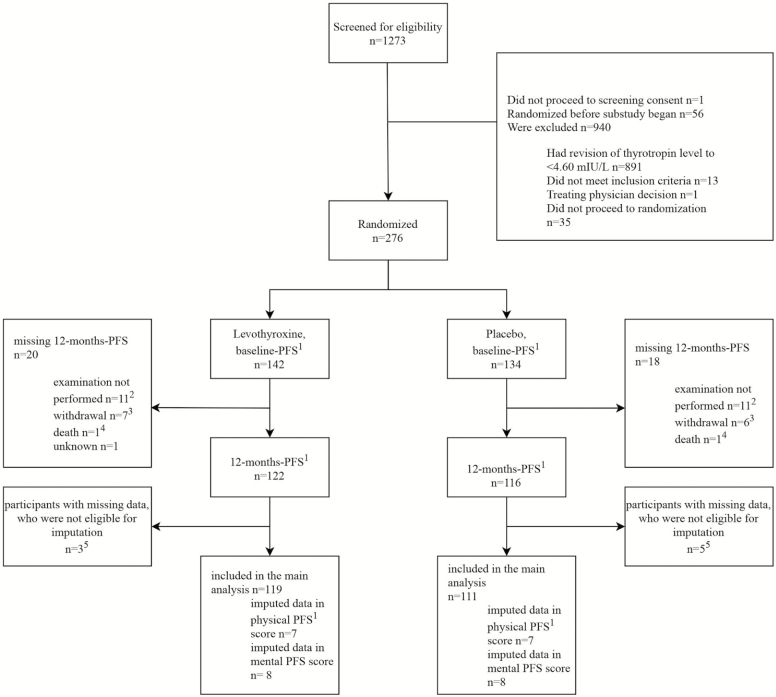
Randomization and follow-up. ^1^The Pittsburgh Fatigability Scale (PFS) has two separate subscores for mental and physical fatigability, each ranging from 0 to 50 with higher scores indicating greater fatigability. ^2^Eleven participants in the levothyroxine group and 11 participants in the placebo group had a follow-up visit but did not answer the PFS-questions due to administrative reasons, *n* = 1 participant in the placebo group had a follow-up visit but did not answer the PFS-questions due to unknown reasons. These missing outcomes were accounted for in sensitivity analysis (Inverse probability weighting [IPW] analysis). ^3^Thirteen participants withdrew, in eight cases due to participants’ decision, in three cases due to adverse events, in one case due to physician recommendation for unknown reason and in one participant the reason is unknown. ^4^One participant died from a septic shock due to a colon perforation combined with a segmental pulmonary embolism left and one participant died from dehydration due to an aspiration pneumonia with acute hypoxemia and progression of cell carcinoma of the hypopharynx. Both deaths were not related to the medication. ^5^Participants with more than three missing questions or lacking information whether the activity has been done or not for a missing answer, as defined in the rules to analyze PFS (13,18).

The study flowchart is shown in Figure 1. Participants were enrolled from January 2014 to December 2015. The last participant completed the study follow-up in November 2016. From 1,273 participants screened, most reverted to normal TSH before randomization and 276 participants were randomized (*n* = 142 allocated to LT4) (6). Fifty-six participants were randomized before the present nested study began. Overall, 38 participants had missing 12-month PFS. Thirteen withdrew from the study, two participants died, one participant did not attend follow-up for unknown reasons, and 22 participants attended the follow-up visit but did not answer the PFS questions (Figure 1). These participants with missing 12-month PFS were similar in baseline physical and mental fatigability as well as age, BMI, number of comorbidities, and the median number of concomitant medications to the participants who were included. Of the 238 participants who had follow-up data, 22 (10 from the LT4 group, 12 from the placebo group) participants had missing answers in the baseline or/and the follow-up PFS physical subscore, of these, eight participants (three in the LT4 group, five in the placebo group) were not imputed because of more than three missing answers, while the remaining 14 were imputed. This resulted in a modified intention-to-treat population of 119 participants in the LT4 and 111 in the placebo group, for both subscores.

Table 1 summarizes the baseline characteristics from all randomized participants (*n* = 276). Characteristics were well balanced between the two groups except for diabetes mellitus (*p* = .12), which was more prevalent in the LT4 group. In the LT4 group, 17 (12.0%) participants started with a lower dose of 25 µg. The mean LT4 dose in the LT4 group was 47 µg (ranging from 25 µg to 50 µg) at baseline and 48 µg (range 0 µg to 100 µg) at 1-year follow-up. The mean ± *SD* TSH was 6.2 ± 1.9 mIU/L at baseline and decreased to 3.1 ± 1.3 in the LT4 group versus 5.3 ± 2.3 in the placebo group (*p* < .001) after 1 year (Table 2).

**Table 1.** Baseline Characteristics of all Participants Randomized (*n* = 276)

Characteristic	Placebo (*N* = 134)	Levothyroxine (*N* = 142)
Age (y)		
Mean ± *SD*	73.5 ± 6.3	73.9 ± 5.1
Range	65.1–92.9	65.3–88.6
Female sex—no. (%)	60 (44.8)	62 (43.4)
White race—no. (%)^a^	132 (99)	141 (99)
Standard housing—no. (%)^b^	130 (97)	140/142 (99)
Weight <50 kg—no. (%)	3 (2)	3 (2)
Body-mass index, kg/m^2^, mean ± *SD*^c^	27.5 ± 4.6	28.0 ± 4.6
Previous medical conditions and clinical descriptors—no. (%)		
Ischemic heart disease^d^	13 (9.7)	14 (9.9)
Atrial fibrillation	16 (11.9)	15 (10.7)
Hypertension	8 (6.0)	4 (2.8)
Diabetes mellitus^e^	14 (10.4)	24 (16.9)
Osteoporosis	17 (12.7)	14 (10.1)
Current smoking^f^	12 (9.0)	8 (5.6)
No. of concomitant medicines—median (IQR^g^)	4 (2–6)	5 (3–5.5)
Laboratory results		
Thyrotropin—mIU/L, mean ± *SD*	6.31 ± 2.00	6.12 ± 1.90
Median (IQR^g^)	5.70 (5.02–6.79)	5.60 (5.04–6.80)
Range	4.60–17.00	4.62–16.76
Free thyroxine—pmol/L, mean ± *SD*	13.5 ± 1.9	13.7 ± 2.0
Levothyroxine dose of 25 μg at randomization (%)	14 (10.5)	17 (11.9)

*Notes*: ^a^Race was reported by the participants.

^b^Standard housing was defined as non-sheltered community accommodation, whereas sheltered housing means a purposed-built grouped housing for older persons.

^c^Calculated as weight (kilograms) divided by squared height (meters).

^d^Defined as history of angina pectoris or previous myocardial infarction.

^e^ *p* = .12.

^f^Defined as current smoking at the time of baseline exam.

^g^IQR denotes interquartile range.

**Table 2.** Thyroid Function, Physical, and Mental Fatigability at Baseline and After 1 Year

Variable	Baseline		At 1 year			
	Levothyroxine (*N* = 119)	Placebo (*N* = 111)	Levothyroxine (*N* = 119)	Placebo (*N* = 111)		
Thyrotropin—mIU/L	6.08 ± 1.80	6.29 ± 2.01	3.08 ± 1.32	5.30 ± 2.34		
Median (IQR)	5.56 (5.04 to 6.62)	5.72 (5.06 to 6.76)	2.95 (2.26 to 3.77)	4.8 (3.62 to 6.57)		
Primary Outcomes					Adjusted Between-Group Difference (95% CI)	*p* Value
PFS physical score (*SD*)^a^	14.7 ± 9.3	11.1 ± 9.1	14.8 ± 9.6	12.4 ± 9.3	0.2 (−1.8 to 2.1)	.88^b^
PFS mental score (*SD*)^a^	7.4 ± 8.0	5.1 ± 6.9	6.0 ± 7.8	6.0 ± 8.0	−1.0 (−2.8 to 0.8)	.26^b^
Secondary Outcomes					Adjusted Odds Ratio (95% CI)	*p* Value
Participants with higher physical fatigability (%)^c^	55 (46.2)	33 (29.7)	52 (43.7)	41 (36.9)	1.0 (0.5 to 1.8)	.88^d^
Participants with higher mental fatigability (%)^c^	27 (22.7)	14 (12.6)	19 (16.0)	20 (18.0)	0.6 (0.3 to 1.4)	.23^d^

^a^The Pittsburgh Fatigability Scale (PFS) physical and mental subscores range from 0 to 50 with higher scores indicating greater fatigability. Crude means are reported.

^b^ *p*-value generated through multiple linear regression model for the follow-up scores adjusted for PFS baseline scores, sex, country and starting levothyroxine dose.

^c^The cut points for higher fatigability are set at ≥15 points for the physical PFS subscore and at ≥13 points for the mental PFS subscore (13,15), as previously established.

^d^ *p*-value generated through multiple logistic regression model for the number of participants with higher fatigability at follow-up adjusted for number of participants with higher fatigability at baseline, sex, country and starting levothyroxine dose.

### Fatigability

Baseline perceived physical and mental fatigability between the LT4 and the placebo group differed by 3.6 points (*p* = .003) and 2.3 points (*p* = .023), respectively (Table 2). Baseline fatigability imbalance was not associated with baseline diabetes imbalance.

The mean follow-up PFS physical score was 14.8 ± 9.6 in the LT4 group and 12.4 ± 9.3 in the placebo group with an adjusted between-group difference of 0.2 (95% CI −1.8 to 2.1, *p* = .88). The mean follow-up PFS mental score was 6.0 ± 7.8 in the LT4 and 6.0 ± 8.0 in the placebo group, respectively, with an adjusted between-group difference of −1.0 (95% CI −2.8 to 0.8, *p* = .26) (Table 2). Fit of the linear regression model was good.

Secondary analysis for binary outcomes distinguishing between participants with lower fatigability and those with higher fatigability (ie, PFS as a binary categorical variable) resulted in nonsignificant findings, with an odds ratio of 1.0 (95% CI 0.5 to 1.8) for the physical fatigability and an odds ratio of 0.6 (95% CI = 0.3 to 1.4) for the mental fatigability (Table 2).

Sensitivity analyses in the participants with complete data did not find a significant difference between the groups (Supplementary Table 1). In order to account for the participants not having 12-month PFS, we conducted sensitivity analyses using inverse probability weighting, and the results remained robust. Sensitivity analyses in the per-protocol population did not find a significant difference between the groups (Supplementary Table 1). Furthermore, including only participants with higher fatigability at baseline did not result in significant between-group differences either. Because of the baseline imbalance in diabetes, we performed sensitivity analyses adjusting for the presence of diabetes and excluding participants with diabetes and the results did not change. Also, performing sensitivity analyses using square-root transformed data did not show benefit of LT4 replacement. In a population limited to participants with TSH levels in the upper quartile (≥6.76 mIU/L), findings were similar.

## Discussion

This nested study within a randomized, placebo-controlled, parallel-group, double-blind multicenter trial in 230 participants aged ≥ 65 years with SHypo did not show a significant benefit of LT4 replacement on physical or mental fatigability after 1 year of treatment, using a more sensitive scale (10) and assessing both, physical and mental fatigability, instead of global fatigue as measured in the main TRUST trial (6).

Previous data on the impact of LT4 on fatigue are limited and conflicting. Even though fatigue is a very common symptom in SHypo and often leads to LT4 prescription (4,5), a recent systematic review showed that large randomized controlled trials investigating the benefit of LT4 replacement on fatigue in SHypo are lacking (8). The only trial, which provided quantitative data for the outcome of fatigue/tiredness, was the TRUST trial. Using the ThyPRO questionnaire to assess tiredness (seven items), the TRUST trial did not find a benefit of LT4 replacement on tiredness in older adults with SHypo. Nevertheless, results were in contrast to the findings of Razvi et al. who reported a benefit of LT4 replacement in SHypo (7). In their randomized, crossover trial in 100 participants with SHypo (mean age 53.8 ± 12.0 years), the authors reported that the proportion of participants with tiredness decreased from 89% to 78% under LT4 replacement, but did not provide measures of precision (such as confidence intervals) and how tiredness was assessed. However, their study differs from ours with respect to the lower mean age of participants and the higher daily LT4 dose (100 μg vs a mean dose of 47 µg at baseline in our study). It would be interesting to compare the fatigability levels of our study population to the fatigability level of a comparable population of older adults, but there are very limited population-based data currently available for comparison because our study was early in using the PFS.

Strengths and novelty of our study were the use of a validated scale that measures the novel construct of fatigability and the consideration of both the physical and mental dimensions of fatigability (10,13). Prevalence of global fatigue varies widely depending on measurement tools, population characteristics, and cutoff points chosen to distinguish between fatigued and non-fatigued participants (21,22). It is, therefore, desirable to bring more objectivity into tiredness evaluation. Fatigability is a concept that classifies fatigue in relation to a defined activity of a specific duration and intensity (10). This conceptualization might lead to a less biased and more objective measure of fatigue than a global fatigue score (13). The classification of fatigue in relation to specific activities is especially helpful in older adults, as these individuals tend to adjust their activity level (eg, by slowing down or shortening the task duration) in order to maintain their perceived fatigue in a tolerable range (13). It is thus possible that two individuals differing in their daily activities (with one having a very active lifestyle and the other a sedentary way of living) report the same tiredness level for the last month. The construct of fatigability is adapted to take the bias of “self-pacing” into account (10,13). The PFS is furthermore the only scale taking into account the multidimensionality of fatigability by distinguishing between mental and physical fatigability (23). Mental fatigability—or self-reported cognitive tiredness related to specific activities—is rarely recognized by the medical community (24). Thus, this study is the first to test the effect of LT4 treatment on both physical and mental fatigability in older subjects with SHypo.

This study has limitations. First, participants were included independent of their baseline fatigability, leading to a proportion of 38% with higher physical fatigability (≥15 points) and 18% with higher mental fatigability (≥13 points). It is possible that findings could differ in a population of (highly) fatigued participants. A sensitivity analysis, including only these participants with higher fatigability, did not reveal a reduction in fatigability level after treatment. Second, 14% (*n* = 38) of participants did not have 12-month PFS measurements. However, these participants had similar characteristics to the participants who did have 12-month PFS and the proportion was balanced between the groups. In particular, these subjects had baseline scores in physical and mental fatigability that did not differ from the participants included in the main analysis. Furthermore, results were robust in a sensitivity analysis using inverse probability weighting to account for possible bias due to missing outcomes (19). Third, the scores for physical and mental fatigability were unbalanced between the two groups at baseline. As analyses were adjusted for baseline scores, this imbalance at baseline should not be expected to impact the results. Fourth, only participants ≥ 65 years were included. Fifth, participants with TSH levels >10 mIU/L accounted for 4% of the study population. Thus, the findings may not be generalizable to individuals with TSH levels >10 mIU/L. Finally, the mean TSH level in the LT4 replacement group was 2.95 mIU/L after 1 year of treatment. We could not exclude that fatigability would have decreased more under a more aggressive LT4 regimen, but potentially at the cost of harms from overtreatment, such as atrial fibrillation or fractures (25–28).

## Conclusion

Over a 1-year follow-up, normalization of TSH levels through LT4 replacement in people aged ≥ 65 years did not show a benefit on perceived physical and mental fatigability compared to placebo. The same finding was shown for participants with higher fatigability at baseline. In line with the findings from the TRUST trial on global fatigue (6), which was the largest randomized controlled trial on the treatment of SHypo, our results do not provide evidence in favor of LT4 replacement to reduce fatigability or fatigue in older adults with SHypo.

## Funding

This nested study on fatigability within the TRUST trial was funded by a grant from the Swiss National Science Foundation (SNSF 320030-172676 to N.R.). The TRUST Thyroid trial was supported by research grant (278148) from the European Union FP7-HEALTH-2011 program and by grants from the Swiss National Science Foundation (SNSF 320030-150025 and 320030-172676 to N.R.) and the Swiss Heart Foundation and Velux Stiftung (grant 974a to N.R). M.R.B’s work was supported by a grant from the Swiss National Science Foundation (P2BEP3_175289). T-H.C.’s research is supported by grants from the Swiss National Science Foundation (PZ00P3-167826). The study medication (levothyroxine and matching placebo) was supplied free of charge by Merck KGaA, Darmstadt, Germany. Merck played no role in the design, analysis, or reporting of the trial. The main TRUST sponsor (NHS Greater Glasgow and Clyde Health Board) contributed to the writing of the protocol.

## Conflict of Interest

None reported.

## Supplementary Material

glaa123_suppl_Supplementary_Table

## Notes

Trial Registration: This nested study is registered on ClinicalTrials.gov (number NCT02500342) with the title “Effect of Thyroid Hormone Replacement on Fatigability in Untreated Older Adults With Subclinical Hypothyroidism (TRUST FATIGUE).” (https://clinicaltrials.gov/ct2/show/NCT02500342?term=fatigue&cond=Subclinical+hypothyro%C3%AFdism&rank=1)

## References

[1] Rugge JB, Bougatsos C, Chou R Screening and treatment of thyroid dysfunction: an evidence review for the U.S. Preventive Services Task Force. Ann Intern Med. 2015;162:35–45. doi:10.7326/M14-1456 25347444

[2] Canaris GJ, Manowitz NR, Mayor G, Ridgway EC The Colorado thyroid disease prevalence study. Arch Intern Med. 2000;160:526–534. doi:10.1001/archinte.160.4.526 10695693

[3] Garber JR, Cobin RH, Gharib H, et al.; American Association of Clinical Endocrinologists and American Thyroid Association Taskforce on Hypothyroidism in Adults Clinical practice guidelines for hypothyroidism in adults: cosponsored by the American Association of Clinical Endocrinologists and the American Thyroid Association. Endocr Pract. 2012;18:988–1028. doi:10.4158/EP12280.GL 23246686

[4] Allport J, McCahon D, Hobbs FD, Roberts LM Why are GPs treating subclinical hypothyroidism? Case note review and GP survey. Prim Health Care Res Dev. 2013;14:175–184. doi:10.1017/S1463423612000230 23174158

[5] Cooper R, Pinkney J, Ayling RM Appropriateness of prescribing thyroxine in primary care. Ann Clin Biochem. 2015;52(Pt 4):497–501. doi:10.1177/0004563214568686 25575699

[6] Stott DJ, Rodondi N, Bauer DC; TRUST Study Group Thyroid hormone therapy for older adults with subclinical hypothyroidism. N Engl J Med. 2017;377:e20. doi:10.1056/NEJMc1709989 28976862

[7] Razvi S, Ingoe L, Keeka G, Oates C, McMillan C, Weaver JU The beneficial effect of L-thyroxine on cardiovascular risk factors, endothelial function, and quality of life in subclinical hypothyroidism: randomized, crossover trial. J Clin Endocrinol Metab. 2007;92:1715–1723. doi:10.1210/jc.2006-1869 17299073

[8] Feller M, Snel M, Moutzouri E, Bauer DC, de Montmollin M, Aujesky D, et al Association of thyroid hormone therapy with quality of life and thyroid-related symptoms in patients with subclinical hypothyroidism a systematic review and meta-analysis. J Am Med Assoc. 2018;320(13):1349–1359. doi:10.1001/jama.2018.13770 PMC6233842 30285179

[9] Watt T, Hegedüs L, Groenvold M, et al. Validity and reliability of the novel thyroid-specific quality of life questionnaire, ThyPRO. Eur J Endocrinol. 2010;162:161–167. doi:10.1530/EJE-09-0521 19797502

[10] Eldadah BA Fatigue and fatigability in older adults. PM R. 2010;2:406–413. doi:10.1016/j.pmrj.2010.03.022 20656622

[11] Peeters RP Subclinical hypothyroidism. N Engl J Med. 2017;376:2556–2565. doi:10.1056/NEJMcp1611144 28657873

[12] LeFevre ML, Force UPST. Screening for thyroid dysfunction: US Preventive Services Task Force Recommendation. Ann Intern Med. 2015;162:641–650. doi:10.7326/M15-0483 25798805

[13] Glynn NW, Santanasto AJ, Simonsick EM, et al. The Pittsburgh Fatigability scale for older adults: development and validation. J Am Geriatr Soc. 2015;63:130–135. doi:10.1111/jgs.13191 25556993 PMC4971882

[14] Stott DJ, Rodondi N, Kearney PM, et al.; TRUST Study Group Thyroid hormone therapy for older adults with subclinical hypothyroidism. N Engl J Med. 2017;376:2534–2544. doi:10.1056/NEJMoa1603825 28402245

[15] Simonsick EM, Schrack JA, Santanasto AJ, Studenski SA, Ferrucci L, Glynn NW Pittsburgh fatigability scale: one-page predictor of mobility decline in mobility-intact older adults. J Am Geriatr Soc. 2018;66:2092–2096. doi:10.1111/jgs.15531 30315707 PMC6322394

[16] Wasson E, Rosso AL, Santanasto AJ, et al.; LIFE Study Group Neural correlates of perceived physical and mental fatigability in older adults: a pilot study. Exp Gerontol. 2019;115:139–147. doi:10.1016/j.exger.2018.12.003 30528639 PMC6331252

[17] Gmelin TSA, Boudreau R, Albert S, Newman A, Venditti E, Glynn N A lifestyle intervention in older adults improves physical fatigability but not mental fatigability. Innovation in Aging. 2018;2(Issue suppl_1):890. doi:10.1093/geroni/igy031.3318

[18] Cooper R, Popham M, Santanasto AJ, Hardy R, Glynn NW, Kuh D Are BMI and inflammatory markers independently associated with physical fatigability in old age? Int J Obes (Lond). 2019;43:832–841. doi:10.1038/s41366-018-0087-0 29795469 PMC6477893

[19] Hernán MA, Lanoy E, Costagliola D, Robins JM Comparison of dynamic treatment regimes via inverse probability weighting. Basic Clin Pharmacol Toxicol. 2006;98:237–242. doi:10.1111/j.1742-7843.2006.pto_329.x 16611197

[20] Pérez LM, Roqué M, Glynn NW, et al. Validation of the Spanish version of the Pittsburgh Fatigability Scale for older adults. Aging Clin Exp Res. 2019;31:209–214. doi:10.1007/s40520-018-0959-0 29736892 PMC6222011

[21] Alexander NB, Taffet GE, Horne FM, et al. Bedside-to-Bench conference: research agenda for idiopathic fatigue and aging. J Am Geriatr Soc. 2010;58:967–975. doi:10.1111/j.1532-5415.2010.02811.x 20722821 PMC4540791

[22] Moreh E, Jacobs JM, Stessman J Fatigue, function, and mortality in older adults. J Gerontol A Biol Sci Med Sci. 2010;65:887–895. doi:10.1093/gerona/glq064 20418349

[23] Ream E, Richardson A Fatigue: a concept analysis. Int J Nurs Stud. 1996;33:519–529. doi:10.1016/0020-7489(96)00004-1 8886902

[24] Lin F, Roiland R, Heffner K, Johnson M, Chen DG, Mapstone M Evaluation of objective and perceived mental fatigability in older adults with vascular risk. J Psychosom Res. 2014;76:458–464. doi:10.1016/j.jpsychores.2014.04.001 24840140 PMC4033905

[25] Somwaru LL, Arnold AM, Joshi N, Fried LP, Cappola AR High frequency of and factors associated with thyroid hormone over-replacement and under-replacement in men and women aged 65 and over. J Clin Endocrinol Metab. 2009;94:1342–1345. doi:10.1210/jc.2008-1696 19126628 PMC2682480

[26] Baumgartner C, da Costa BR, Collet TH, et al.; Thyroid Studies Collaboration Thyroid Function Within the Normal Range, Subclinical Hypothyroidism, and the Risk of Atrial Fibrillation. Circulation. 2017;136:2100–2116. doi:10.1161/CIRCULATIONAHA.117.028753 29061566 PMC5705446

[27] Blum MR, Bauer DC, Collet TH, et al.; Thyroid Studies Collaboration Subclinical thyroid dysfunction and fracture risk: a meta-analysis. JAMA. 2015;313:2055–2065. doi:10.1001/jama.2015.5161 26010634 PMC4729304

[28] Pearce SH, Brabant G, Duntas LH, et al. 2013 ETA Guideline: management of subclinical hypothyroidism. Eur Thyroid J. 2013;2:215–228. doi:10.1159/000356507 24783053 PMC3923601

