# Supplementary material for: Effect of Thyroid Hormone Therapy on Fatigability in Older Adults With Subclinical Hypothyroidism: A Nested Study Within a Randomized Placebo-Controlled Trial
Source: J Gerontol A Biol Sci Med Sci. 2020 Jun 6;75(9):e89–94. doi: 10.1093/gerona/glaa123 (PMC7494024; doi:10.1093/gerona/glaa123)
Supplement: glaa123_suppl_Supplementary_Table [file glaa123_suppl_supplementary_table.docx]

Supplemental Table 1

| Variable | Baseline | | | | At 1 year | | | | | |
| --- | --- | --- | --- | --- | --- | --- | --- | --- | --- | --- |
|  | Levothyroxine | | Placebo | | Levothyroxine | | Placebo | | Adjusted Between-group Difference (95% CI) | P Value |
|  | N | Mean±SD | N | Mean±SD | N | Mean±SD | N | Mean±SD |  |  |
| Participants with higher fatigability at baseline^1^ |  |  |  |  |  |  |  |  |  |  |
| PFS physical score (SD) (n=88)^2^ | 55 | 23.2±6.1 | 33 | 22.2±7.3 | 55 | 19.2±9.1 | 33 | 20.0±9.8 | -0.4 (-3.6 to 2.8)^2^ | 0.79^3^ |
| PFS mental score (SD) (n=41) | 27 | 20.0±5.7 | 14 | 19.5±7.5 | 27 | 9.8±9.9 | 14 | 15.4±12.2 | -2.2 (-8.8 to 4.5)^2^ | 0.51^3^ |
| Participants with complete outcome data^5^ |  |  |  |  |  |  |  |  |  |  |
| Complete data for PFS physical score (SD) (n=116) | 112 | 15.1±9.0 | 104 | 11.5±9.1 | 112 | 14.5±9.7 | 104 | 12.2±9.3 | -0.3 (-2.1 to 1.6)^2^ | 0.77^3^ |
| Complete data for PFS mental (SD) score (n=114) | 111 | 7.6±8.0 | 103 | 5.3±7.0 | 111 | 5.6±7.7 | 103 | 5.8±7.8 | -1.2 (-3.0 to 0.6)^2^ | 0.18^3^ |
| Per protocol analysis^6^ |  |  |  |  |  |  |  |  |  |  |
| PFS physical score (SD) (n=205) | 104 | 14.1±9.2 | 101 | 10.8±8.7 | 104 | 14.5±9.4 | 101 | 12.2±8.8 | 0.2 (-1.8 to 2.2)^2^ | 0.86^3^ |
| PFS mental score (SD) (n=205) | 104 | 7.5±8.0 | 101 | 5.0±6.6 | 104 | 5.6±6.8 | 101 | 5.9±7.8 | -1.4 (-3.2 to 0.4)^2^ | 0.13^3^ |

| Excluding participants with diabetes mellitus |  |  |  |  |  |  |  |  |  |  |
| --- | --- | --- | --- | --- | --- | --- | --- | --- | --- | --- |
| PFS physical score (SD) (n=200) | 98 | 13.9±9.1 | 102 | 11.1±9.2 | 98 | 13.8±9.2 | 102 | 12.8±9.3 | -0.7 (-2.7 to 1.2)^2^ | 0.46^3^ |
| PFS mental score (SD) (n=200) | 98 | 7.3±7.5 | 102 | 5.1±6.8 | 98 | 5.9±7.7 | 102 | 6.1±8.2 | -1.2 (-3.2 to 0.7)^2^ | 0.20^3^ |
| Adjusting for diabetes mellitus |  |  |  |  |  |  |  |  |  |  |
| PFS physical score (SD) (n=230) | 119 | 14.7±9.3 | 111 | 11.1 ±9.1 | 119 | 14.8±9.6 | 111 | 12.4±9.3 | 0.1 (-1.9 to 2.0)^4^ | 0.95^5^ |
| PFS mental score (SD) (n=230) | 119 | 7.4±8.0 | 111 | 5.1±6.9 | 119 | 6.0±7.8 | 111 | 6.0±8.0 | -1.0 (-2.9 to 0.8)^4^ | 0.26^5^ |
| Sqrt transformed data analysis^6^ |  |  |  |  |  |  |  |  |  |  |
| PFS physical score (SD) (n=230) | 119 | 3.6±1.4 | 111 | 3.0±1.5 | 119 | 3.6±1.4 | 111 | 3.2±1.5 | 0.1 (-0.2 to 0.4)^2^ | 0.61^3^ |
| PFS mental score (SD) (n=230) | 119 | 2.2±1.6 | 111 | 1.7±1.5 | 119 | 1.9±1.6 | 111 | 1.8±1.7 | -0.1 (-0.5 to 0.2)^2^ | 0.49^3^ |
| IPW-analysis^7^ |  |  |  |  |  |  |  |  |  |  |
| PFS physical score (SD) (n=230) | 119 | 14.7±9.3 | 111 | 11.1 ±9.1 | 119 | 14.9±9.6^8^ | 111 | 12.4±9.3^8^ | -0.75 (-2.7 to 1.2)^2^ | 0.45^3^ |
| PFS mental score (SD) (n=230) | 119 | 7.4±8.0 | 111 | 5.1±6.9 | 119 | 6.0±7.8^8^ | 111 | 6.0±7.9^8^ | 0.26 (-1.6 to 2.1)^2^ | 0.78^3^ |
| Participants with TSH values in the upper quartile^9^ |  |  |  |  |  |  |  |  |  |  |
| PFS physical score (SD) (n=56) | 28 | 11.5±8.5 | 28 | 6.3±6.3 | 28 | 13.0±8.5 | 28 | 10.3±7.5 | -0.01 ( -4.2 to 4.2) | 0.10^3^ |
| PFS mental score (SD) (n=56) | 28 | 5.5±7.7 | 28 | 3.4±4.1 | 28 | 6.1±8.7 | 28 | 4.1±5.1 | 0.62 ( -2.8 to 4.0) | 0.72^3^ |

If not otherwise indicated, the crude means are reported. The Pittsburgh Fatigability Scale (PFS) physical and mental subscores range from 0-50 with higher scores indicating greater fatigability.

^1^ Including only participants with higher fatigability at baseline (≥15 points in the PFS physical or ≥13 points in the PFS mental score)

^2^ Between-group differences generated through multiple linear regression model for the follow-up scores adjusted for PFS baseline scores, sex, country and starting levothyroxine dose

^3^ P value generated through multiple linear regression model for the follow-up scores adjusted for PFS baseline scores, sex, country and starting levothyroxine dose.

^4^ Between-group differences generated through multiple linear regression model for the follow-up scores adjusted for PFS baseline scores, sex, country, starting levothyroxine dose and presence of diabetes mellitus

^5^ P value generated through multiple linear regression model for the follow-up scores adjusted for PFS baseline scores, sex, country, starting levothyroxine dose and presence of diabetes mellitus.

^6^ Square root transformation was performed prior to multiple linear regression model for the follow-up scores adjusted for the same variables as in the main analysis in order to correct for skewed distributions.

^7^ Inverse probability weighting (IPW) was performed in order to correct for missing data at 12 months follow-up. The covariables included in the logistic regression model to estimate IPWs were BMI, age, country, sex and the number of comorbidities.

^8^ Weighted means (IPW)

^9^ Including only participants with TSH levels in the upper quartile (≥ 6.76 mIU/L)
